# Anti-Oxidant and Anti-Enzymatic Activities of Sea Buckthorn (*Hippophaë rhamnoides* L.) Fruits Modulated by Chemical Components

**DOI:** 10.3390/antiox8120618

**Published:** 2019-12-04

**Authors:** Karolina Tkacz, Aneta Wojdyło, Igor Piotr Turkiewicz, Łukasz Bobak, Paulina Nowicka

**Affiliations:** 1 Department of Fruit, Vegetable and Nutraceutical Plant Technology, The Faculty of Biotechnology and Food Science, Wrocław University of Environmental and Life Sciences, 37 Chełmońskiego Street, 51-630 Wrocław, Poland; karolina.tkacz@upwr.edu.pl (K.T.); igor.turkiewicz@upwr.edu.pl (I.P.T.); paulina.nowicka@upwr.edu.pl (P.N.); 2 Department of Animal Products Technology and Quality Management, The Faculty of Biotechnology and Food Science, Wrocław University of Environmental and Life Sciences, 37 Chełmońskiego Street, 51-630 Wrocław, Poland; lukasz.bobak@upwr.edu.pl

**Keywords:** sea buckthorn berries, 2,2’-Azobis(2-amidinopropane)dihydrochloride (ABTS), ferric reducing ability of plasma (FRAP), oxygen radical absorbance capacity (ORAC), *α*-amylase, *α*-glucosidase, lipase, lipoxygenase, fatty acids, vitamins

## Abstract

The aim of this study was to analyze in vitro biological activities as anti-oxidant, anti-*α*-amylase, anti-*α*-glucosidase, anti-lipase, and anti-lipoxygenase activity, relative to bioactive components (phenolic acids, flavonols, xanthophylls, carotenes, esterified carotenoids, tocopherols, tocotrienols, and fatty acids) and the basic chemical composition (sugars, organic acid, dry matter, soluble solid, pH, titratable acidity, ash, pectins, and vitamin C) of *Hippophaë rhamnoides* berries. Six sea buckthorn cultivars commonly grown in Poland were analyzed including Aromatnaja, Botaniczeskaja-Lubitelskaja, Józef, Luczistaja, Moskwiczka, and Podarok Sadu. Berries contained 1.34–2.87 g of sugars and 0.96–4.22 g of organic acids in 100 g fresh weight, 468.60–901.11 mg of phenolic compounds, and 46.61–508.57 mg of carotenoids in 100 g dry mass. The fatty acid profile was established: palmitic > palmitoleic > oleic and linoleic > stearic and linolenic acids. The highest anti-oxidant (34.68 mmol Trolox/100 g dry mass) and anti-*α*-amylase potential (IC_50_ = 26.83 mg/mL) was determined in Aromatnaja, anti-*α*-glucosidase in Botaniczeskaja-Lubitelskaja (IC_50_ = 41.78 mg/mL), anti-lipase in Moskwiczka and Aromatnaja (average IC_50_ = 4.37 mg/mL), and anti-lipoxygenase in Aromatnaja and Podarok Sadu fruits (100% inhibition). The studied sea buckthorn berries may be a raw material for the development of functional foods and nutraceutical products rich in compounds with high biological activity.

## 1. Introduction

Sea buckthorn (*Hippophaë rhamnoides* L.) is a thorny, deciduous shrub belonging to the Elaeagnaceae family. Six species of *Hippophaë* and 12 subspecies are currently recognized, including ssp. *sinensis*, ssp. *mongolica*, and ssp. *rhamnoides*, which are the most economically and commercially important. There are over 150 cultivars of sea buckthorn, but new thornless and easier-to-harvest varieties are still being selected. Sea buckthorn naturally grows on sea coasts and river valleys of Central and Northern Europe, Russia, China, Mongolia, Central Asia, and slopes of the Caucasus and Himalayas. The plant is cultivated mainly in the Northern Hemisphere and its largest producer is China [1,2,3,4].

Fleshy and soft sea buckthorn fruits are yellow, orange, or red, round or oblong, and 6–15 mm in diameter [5]. Due to the similarity of berries, the plant is commonly confused with scarlet firethorn (*Pyracantha coccinea*), rock cotoneaster (*Cotoneaster horizontalis*), or rowan (*Sorbus aucuparia*), whose raw fruits are poisonous. The sea buckthorn aroma is compared to strawberries, peach, mango, apricot, papaya, and citrus, but mostly to pineapple, which results from a similar ester profile [6]. In the food industry, sea buckthorn is used as a raw material enriching the pro-health value or increasing the acidity of fruit products. Berries are intended for the production of jams, juices, soft drinks, liqueurs, wine, or as an addition to beers, kefir, and cheeses. By contrast, oil obtained from seeds and pulp is used as a cosmetic and dietary supplement, and is used less frequently as a culinary product. Production residues can be a functional ingredient in meat or animal feed [1,2,5,7].

The therapeutic properties of bark, leaves, and fruits were already known in ancient Greece as well as in Tibetan and Mongolian medicine. In the cosmetics industry, the plant is used in dermatological diseases, hair care, revitalization of wounds and skin burns, and as a form of natural protection against UV-B radiation. The results of previous in vitro and in vivo studies [2,5,7,8,9,10] confirm the effectiveness of sea buckthorn extracts in the prevention of hyperglycemia, hyperinsulinemia, and hyperlipidemia, together with hepatoprotective, anti-carcinogenic, antibacterial, and antifungal effects, as well as positive functioning of the digestive system and eyesight. The properties of sea buckthorn are due to the high concentrations of flavonoids (mainly flavonols), carotenoids (principally β-cryptoxanthin and β-carotene), ascorbic acid, vitamin E (the most active form, α-tocopherol, dominates), and fatty acids (omega-3, omega-6, omega-7, and omega-9) present in seeds, skin, and flesh [6,11,12].

A number of studies have been carried out on sea buckthorn in different world regions, but the knowledge about biologically active compounds and pro-health potential of cultivars grown in Poland is limited. Given the above, the aim of this study was to analyze biological activities (anti-oxidant, anti-α-amylase, anti-α-glucosidase, anti-lipase, and anti-lipoxygenase effects) relative to selected bioactive components (flavonols and phenolic acids, xanthophylls, carotenes, esterified carotenoids, tocopherols and tocotrienols, fatty acids), and the basic chemical composition (sugars, organic acid, dry matter, soluble solid, pH, titratable acidity, ash, pectins, vitamin C) of berries of six commonly grown *H. rhamnoides* cultivars in Poland.

α-Amylase and α-glucosidase break down polysaccharides to glucose. Therefore, their inhibition is one of the methods of postprandial hyperglycemia reduction. This effect plays a key role in treating type 2 diabetes, which, according to WHO, affects 8.5% of the global adult population. In turn, pancreatic lipase breaks down dietary triglycerides into bioavailable forms—fatty acids and glycerol. Its inhibition can reduce energy intake at a meal, which is part of the strategy of overweight and obesity therapy. The lipoxygenase pathway, including lipoxygenase 5-LOX, 12-LOX, and 15-LOX, is associated with the production of hydroperoxy fatty acids and leukotrienes. Increased concentrations of these products correlate with the progression of, inter alia, inflammatory bowel disease, asthmatic bronchitis, rheumatoid arthritis, cancers, and cardiovascular diseases [13].

Therefore, it was assumed that the results will allow the identification of significant differences in the pro-health potential and composition of the studied sea buckthorn cultivars for further use in the design of innovative functional products, nutraceuticals, and cosmeceuticals. Additionally, this study should indicate cultivars with the highest biological potential for further use in planning and expanding cultivations. Furthermore, the results of the cultivar Józef, bred in Poland, are presented for the first time. This creates promising perspectives for commercial production of this sea buckthorn cultivar and use in the food industry as a source of health-promoting substances and antioxidant properties.

## 2. Materials and Methods

### 2.1. Chemicals

Standards of sugars, organic acids, phenolic compounds, and carotenoid compounds were purchased from Extrasynthese (Genay, France), and the rest of the reagents were bought from Merck KgaA (Darmstadt, Germany). The samples before chromatographic analysis were filtered through a Hydrophilic PTFE 0.20 μm membrane (Millex Samplicity Filters, Merck KgaA, Darmstadt, Germany).

### 2.2. Plant Materials

**Figure 1:**
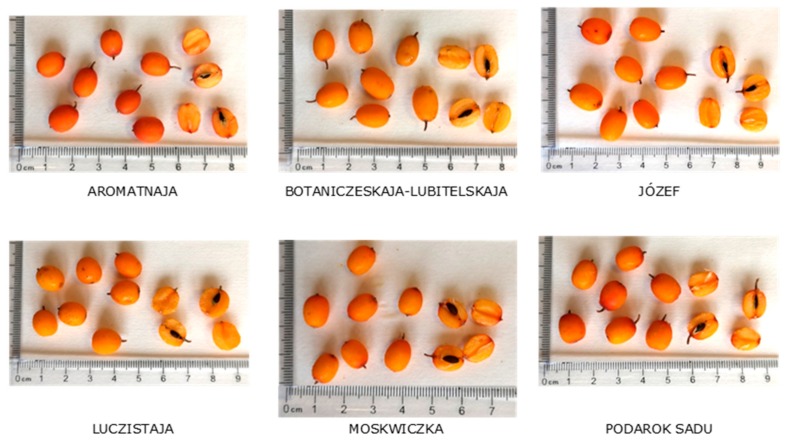
Berries of sea buckthorn cultivars.

The fruits of six sea buckthorn (*Hippophaë rhamnoides* L.) cultivars—Aromatnaja, Botaniczeskaja-Lubitelskaja, Józef, Luczistaja, Moskwiczka, and Podarok Sadu—were tested (Figure 1). Ripe berries were collected in early July and August 2018 from the Research Institute of Horticulture in Skierniewice (Poland). Fresh fruits were used to analyze the basic chemical composition. The second portion of selected berries was frozen, freeze-dried for 24 h (Christ Alpha 1–4 LSC, Martin Christ GmbH, Osterode am Harz, Germany) and crushed by a laboratory mill (A11, IKA, Darmstadt, Germany). The homogeneous materials were stored in a freezer at −80 °C until undergoing the other analysis.

### 2.3. Basic Chemical Composition

The soluble solids content was expressed in °Bx using a digital refractometer (Atago RX-5000, Atago Co. Ltd., Saitama, Japan). The instrument was calibrated using distilled water. Liquid and homogenized raw material was applied to the dry prism surface. The measurement was taken at 20 °C. The dry matter was determined by mixing the sample with diatomaceous earth, pre-drying, and final drying under reduced pressure. Titratable acidity (TA) was analyzed by the titration of homogenous fresh fruits with 0.1N NaOH to pH 8.1 and the result were expressed as g malic acid/100 g FW (fresh weight). TA and pH were determined using an automatic pH titrator system (TitroLine 5000, Xylem Analytics GmbH, Weilheim in Oberbayern, Germany). The soluble solids content, dry matter, and titratable acidity were taken according to European Standards, PN-EN 12143:2000, PN-EN 12145:2001, and PN-EN 12145:2000, respectively. Pectin content (g/100 g FW) was measured according to the Morris method reported by Pijanowski et al. [14]. Ash (%), l-ascorbic acid (mg/100 g FW), sugars, and organic acids contents (g/100 g FW) were determined, as reported previously by Wojdyło et al. [15]. Sugars and organic acids were analyzed using high pressure liquid chromatography including the evaporative light scattering detector (HPLC-ELSD) and ultra performance liquid chromatography-photodiode array detector (UPLC-PDA) methods. All measurements were taken three times.

### 2.4. Analysis of Phenolic Compounds

The extraction of the samples for phenolic compounds and their chromatographic analysis were performed exactly as described by Wojdyło et al. [15]. The samples were analyzed by an Ultra-Performance Liquid Chromatography Photodiode Array Detector (UPLC-PDA; Acquity UPLC System, Waters Corp., Milford, MA, US). The study identified phenolic acids and flavonols, and their sums were calculated as *p*-coumaric acid and isorhamnetin-3-*O*-rutinoside, respectively, which is based on dominant compounds and compared with reference standards. All results were taken in triplicate and shown as mg/100 g DM of berries (dry mass).

### 2.5. Analysis of Carotenoids, Tocopherols, and Tocotrienols

The extraction of the samples for carotenoid compounds was made as previously described by Wojdyło et al. [15] and Nowicka et al. [16]. The determination of carotenoids was made using the equipment as in Section 2.4, according to the protocol given by Wojdyło et al. [15]. The powder samples of fruits (0.20 g) containing 10% MgCO_3_ and 1% butylhydroxytoluene (BHT) to prevent oxidation were continuously shaken with 5 mL of a ternary mixture of methanol/acetone/hexane (1:1:2, *v:v:v*) at 300 rpm (DOS-10L Digital Orbital Shaker, Elmi Ltd., Riga, Latvia) for 30 min in the dark. Recovered supernatants were obtained after 4–5 times being re-extracted of solid residue. All combined fractions collected after centrifugation (4 °C, 7 min at 19,000× *g*, MPW-350, Warsaw, Poland) were evaporated to dryness. The pellet was diluted using 2 mL of 100% methanol, filtered through a hydrophilic polytetrafluoroethylene (PTFE) 0.20-μm membrane (Millex Samplicity Filter, Merck, Darmstadt, Germany) and used for analysis.

Carotenoids were carried out on an ACQUITY UPLC BEH RP C18 column being protected by the guard column of the same materials (1.7 mm, 2.1 mm 100 mm, Waters Corp., Milford, MA, USA) operated at 30 °C. The elution solvents were linear gradient of acetonitrile:methanol (70:30, *v:v*) (A) and 0.1% formic acid (B). The runs were monitored at 450 nm. The photodiode array detector PDA spectra were measured over the wavelength range of 200–700 nm in steps of 2 nm. The retention times and spectra were compared to those of the authentic standards. All incubations were done in triplicate. The results were expressed as mg per kg of dm. Samples for the analysis of tocopherols and tocotrienols were prepared as follows. The fresh sea buckthorn berries (∼3g) were homogenized with two times as much of the ethanol portion mixed with 0.05% butylated hydroxytoluene (BHT). Saponification was carried out using 60% CaOH, at a temperature of 50 °C for 2 h. Then, the samples were mixed with hexane:ethyl acetate with 0.05% BHT. After that, NaOH (saturated solution) was added. The upper layer was collected, evaporated, and dissolved in methanol with 0.05% BHT. The solutions were filtered through a Hydrophilic PTFE 0.20 μm membrane and used for UPLC analysis. The analysis of tocopherols and tocotrienols was carried out by using Ultra-Performance Liquid Chromatography with a fluorescence detector (UPLC-FL). The column ACQUITY UPLC BEH RP C18 (1.7 mm, 2.1 mm × 100 mm, Waters Corp., Milford, MA, US) being protected by a guard column of the same materials was operated at 30 °C. Identification and quantification was performed based on reference standards and calibration curves. The samples (5 μL) were injected, and the elution was completed in 12 min with an isocratic method of methanol with water (88:12, *v:v*) flow rates of 0.45 mL/min. All incubations were done in triplicate. The results were expressed as mg per kg of dm. 

### 2.6. Analysis of Fatty Acids

Fatty acids were extracted and tested with the technique of gas chromatography with mass spectrometry (GC-MS), in the same way as described by Nowacki et al. [17]. The samples were analyzed using a GC 6890 gas chromatograph coupled with a 5983 MS mass spectrometer (Agilent Technologies Inc., Santa Clara, CA, US) equipped in a quadrupole mass detector. Measurements were taken in triplicate. The results of fatty acid studies were expressed as the percentage of total fatty acids of sea buckthorn berries.

### 2.7. Determintation of Biological Activity: Anti-Oxidant, Anti-α-Amylase, Anti- α-Glucosidase, Anti-Lipase, and Anti-Lipoxygenase

The extraction procedure was the same for all determinations and was carried out identically, as described by Nowicka et al. [16]. The ABTS, FRAP, and ORAC assays were conducted as previously reported by Re et al. [18], Benzie and Strain [19], and Ou et al. [20], respectively. The ABTS•^+^ (2,2′-azine-bis-(3-ethylene-benzothiazoline-6-sulfonic acid) scavenging test is based on measuring the decrease in the color intensity inversely proportional to the antioxidant content. An ABTS•^+^ solution was prepared with an absorbance of 0.700 ± 0.02 at a wavelength of 734 nm. Sea buckthorn extracts and the ABTS•^+^ solution were mixed and, after 6 min, the absorption at the wavelength above was measured. Distilled water was blank. The results were calculated based on the calibration curve (*R*^2^ = 0.9950) for Trolox concentrations 0.100 to 0.900 mM.

The FRAP method involves determining the ability to reduce Fe^3+^ ions by antioxidant substances contained in sea buckthorn extracts to the blue Fe^2+^ ions complex. Sea buckthorn extracts were mixed with distilled water. The absorbance of the samples was measured 10 min after the addition of the FRAP reagent (acetate buffer, 2,4,6-Tris(2-pyridyl)-s-triazine (TPTZ) in HCl and FeCl_3_ × 6H_2_O in a volume ratio of 10:1:1, *v:v:v*), at a wavelength of 593 nm. The results were calculated based on the calibration curve (*R*^2^ = 0.9899) for Trolox concentrations 0.050 to 0.900 mM.

The analysis of oxygen radical absorbance capacity (ORAC) consists of a spectrofluorometric measurement of the decrease in fluorescence caused by oxidation of a fluorescent substance under the influence of free radicals, but in the presence of antioxidant substances. Samples containing sea buckthorn extract, phosphate buffer, and fluorescein were incubated at 37 °C throughout the analysis period. 2,2’-Azobis(2-amidinopropane)dihydrochloride was added and the spectrofluorometric measurement was performed every 5 min at an excitation wavelength 493 nm and an emission wavelength of 515 nm. The blank was a phosphate buffer. The antioxidant activity of the tested samples was obtained by comparing the surface under the fluorescence decrease curves over time with the surface for pure Trolox solutions (12.5, 25.0, 50.0, and 75.0 µM).

The ABTS, FRAP, and ORAC results were expressed in mmol TE (Trolox)/100 g sample.

The anti-*α*-amylase, anti-*α*-glucosidase, and anti-lipase activity were studied, according to methods reported by Nowicka et al. [16] and Podsędek et al. [21]. Briefly, analysis of the anti-*α*-amylase inhibitory activity is based on a spectrophotometric measurement of the color change as a result of a reaction of iodine in potassium iodide with the remaining starch after enzymatic hydrolysis. Basic samples contained sea buckthorn extracts, starch solution, and *α*-amylase. After incubation at 37 °C, the reaction was stopped using 0.4 M HCl. A solution of potassium iodide with iodine was added. Reference samples contained phosphate buffer instead of an enzyme. The acarbose was included as a positive control and absorbance was measured at 600 nm.

The analysis of *α*-glucosidase inhibitory activity consists of the reaction of the enzyme with a β-d-glucosidase substrate producing a yellow solution upon cleavage. Basic samples containing sea buckthorn extracts and enzymes were incubated as above. After the addition of the substrate, the mixture was incubated again and measurement was made at 405 nm. As in the above analysis, the reference samples contained buffer instead of enzymes and the acarbose was included as a positive control.

The analysis of lipase inhibitory activity is based on a spectrophotometric measurement of the amount of *p*-nitrophenol formed from *p*-nitrophenyl acetate. Basic samples contained sea buckthorn extracts, Tris-HCl buffer, and the enzyme. After 5 min of incubation at 37 °C, the substrate was added. Then incubation continued for 15 min. Reference samples contained buffer instead of the enzyme and the orlistat was used as a positive control. Absorbance was measured at 400 nm. The results of anti-*α*-amylase, anti-*α*-glucosidase, and anti-lipase activity are presented as IC_50_ in mg/mL, i.e., the amount of the sample that is able to reduce enzyme activity by 50%.

Inhibitory activity toward 15-lipoxygenase was measured in accordance with Chung et al. [13]. Basic samples containing sea buckthorn extract and enzymes were incubated at 37 °C. Then, linoleic acid was added and incubation continued for 20 min. The mixture was measured at 210 nm. Reference samples contained Tris-HCl buffer instead of the enzyme. The results were expressed as a percentage inhibition (at the concentration of 30 mg/mL).

All tests: anti-oxidant (ABTS, ORAC, FRAP), anti-α-amylase, anti- α-glucosidase, anti-lipase, and anti-lipoxygenase were performed in triplicate using a microplate reader Synergy^TM^ H1 (BioTek, Winooski, VT, US).

### 2.8. Statistical Analysis

One-way analysis of variance (ANOVA, *p* < 0.05), Tukey’s test, Pearson’s correlation coefficients, and Principal Component Analysis (PCA) were carried out using XLSTAT for Microsoft Excel 2010 (Microsoft Corp., Redmond, WA, US) and Statistica 13.1 (StatSoft, Cracow, Poland). The results were presented as the mean value (*n* = 3) ± standard deviation (SD).

## 3. Results and Discussion

### 3.1. Basic Chemical Composition of Sea Buckthorn Cultivars

Table 1 presents the basic chemical composition of sea buckthorn cultivars: Aromatnaja, Botaniczeskaja-Lubitelskaja, Józef, Luczistaja, Moskwiczka, and Podarok Sadu. Analogous or closely related cultivars have been analyzed by other researchers but were cultivated in other climatic and soil conditions, including Sweden, Belarus, Finland, and Canada. However, this is the first report on the new cultivar Józef, bred in Poland.

Dry matter of *H. rhamnoides* berries was statistically different and ranged from 11.78% (Luczistaja) to 13.08% (Aromatnaja). A soluble solid was examined from 5.7 (Luczistaja) to 7.2 °Bx (Moskwiczka and Aromatnaja) and was lower than indicated in berries cultivated in Canada [22] and Finland [23] (to 12.6 °Bx). Sea buckthorn fruits had a low pH (from 2.89 to 2.95 in the case of Moskwiczka and Aromatnaja, respectively) and titratable acidity (from 2.48 to 2.79 g malic acid/100 g fresh weight (FW) for Moskwiczka and Podarok Sadu, respectively). The obtained results were in line with other reports, according to which pH was from 2.30 to 3.20 and titratable acidity from 2.00 to 4.66 g malic acid/100 g [22,23,24]. Yang [24] stated that pH may be dependent on the harvest period of berries because values increased from late August to mid-October, and then decreased. Additionally, low ash content was determined, from 0.31% (Moskwiczka) to 0.43% (Aromatnaja), compared to data provided by Bal et al. [1], for sea buckthorn berries cultivated in Japan (1.78% and 1.8%). Pectin content was from 0.21% (Józef) to 0.68% (Luczistaja). Aromatnaja berries were characterized by about half the amount of pectins (0.34%) and twice as high vitamin C content (158.81 mg/100 g FW) compared to other examined berries. The new cultivar (cv.) Józef contained a similar amount of vitamin C since the cultivar was the poorest in it (64.92 mg/100 g FW). In studies by Teleszko et al. [12], Aromatnaja fruits were the richest in ascorbic acid (130.97 mg/100 g FW), followed by Podarok Sadu, Moskwiczka, Botaniczeskaja-Lubitelskaja, and Luczistaja (from 82.61 to 52.86 mg/100 g FW). Kawecki et al. [25] reported two to three times more vitamin C content in Podarok Sadu and Botaniczeskaja berries than in our study. Research on sea buckthorn fruits from Germany [26], Finland [23], and Canada [22] reported a similar content of vitamin C as in the studied cultivars, and also proved that the factors determining the vitamin C content are mainly the cultivar, maturity stage, fruit size, and harvest season. Due to the lack of ascorbinase in berries and juice, vitamin C is stable for six days, whereas the annual frozen storage does not change its content [27].

The studied sea buckthorn fruits contained from 1.34 (Botaniczeskaja-Lubitelskaja) to 2.87 g of sugars/100 g FW (Moskwiczka) (Table 1). The most abundant sugar was glucose, which constituted 86.58% to 92.68% of all sugars (in the case of Podarok Sad and Moskwiczka, respectively). Significantly lower concentrations of sorbitol and fructose were determined (maximum 6.36% and 5.88% of total sugars, respectively). Only Luczistaja berries contained more fructose than sorbitol. In Luczistaja, Podarok Sadu, Józef, and Aromatnaja fruits, rhamnose below 3.5% of all sugars was quantitated, while, in other tested fruits, it was not detected. Our results corroborated those published by Kawecki et al. [25], Tiitinen et al. [23], and Zheng et al. [22]. For example, the tested cv. Podarok Sadu contained 1.49% of sugars, compared to an analogous cultivar grown in Poland, tested by Kawacki et al. [25], and containing 1.99%.

The content of organic acids (Table 1) was from 0.96 to 4.22 g/100 g FW (for Moskwiczka and Luczistaja, respectively). In all cultivars, organic acid concentrations were studied in the following order: malic acid (from 63.11% to 85.42% of acids) > quinic acid (from 6.77% to 32.04% of acids) > isocitric acid (to 15.79% of acids) > citric acid (from 0.32% to 4.44% of acids) > oxalic acid (from 0.32% to 2.08% of acids). The exceptions were cv. Moskwiczka, in which no isocitric acid was detected, cv. Józef, which contained more citric acid than isocitric acid, and cv. Aromatnaja, in which more isocitric acid than quinic acid was identified. Furthermore, maleic and shikimic acids were identified, but were in quantities below 0.01 g/100 g FW (results not shown in the table). The newly studied cv. Józef contained the amount of sugars and acids most similar to the average amounts of sugars and organic acids in the tested sea buckthorn cultivars, 1.93 and 2.23 mg/100 g FW, respectively. Studies on sea buckthorn cultivars grown in Canada, Finland, or Poland indicated the organic acid content from 0.96% to 5.40% and the main acids were malic and then quinic [22,23,24,25].

The sugars/organic acids ratio was low and ranged from 0.40 to 2.99 (for Luczistaja and Moskwiczka, respectively). The results point to the need to correct the taste of selected cultivars, in particular those where the ratio was below 1.0, i.e., Botaniczeskaja-Lubitelskaja, Luczistaja, Podarok Sadu, and the new cv. Józef.

### 3.2. Analysis of Phenolic Compounds of Sea Buckthorn Cultivars

Among the phenolic compounds, phenolic acids and flavonols were quantified in tested sea buckthorn fruits and the results are summarized in Table 1. The total content of phenolic compounds in sea buckthorn berries ranged from 468.60 mg (Luczistaja) to 901.11 mg/100 g dry mass (DM) (Moskwiczka). Other reports indicated comparable phenolic compound values in *H. rhamnoides* berries from 385 to 1442 mg/100 g DM [7,28,29]. The tested Podarok Sadu fruits contained half the amount of phenolic compounds compared to the analogous cultivar collected in Belarus and studied by Zadernowski et al. [29].

The concentration of phenolic acids in studied berries was from 5.18 mg (Botaniczeskaja-Lubitelskaja) to 8.94 mg/100 g DM (Podarok Sadu). The cultivar created in Poland—Józef—contained the same amount of organic acids as Aromatnaja (6.11 mg/100 g DM), and these were richer than Botaniczeskaja-Lubitelskaja and Luczistaja berries. Other reports indicated several times higher content of phenolic acids: from 37.9 mg to 443.92 mg/100 g DM [5,7,29]. Podarok Sadu contained 43 times less phenolic acids than the analogous cultivar tested by Zadernowski et al. [29]. Research by Teleszko et al. [12] determined the phenolic acid concentration from 5.81 mg in Avgustinka to 3.11 mg/100 g FW for Luczistaja berries.

In berries of tested cultivars, approximately 98.94% of the total phenolic compounds were flavonols. Their quantity ranged from 463.14 mg to 893.92 mg/100 g DM, and the order of cultivars in terms of flavonols content was as follows: Moskwiczka > Józef > Aromatnaja > Podarok Sadu > Botaniczeskaja-Lubitelskaja > Luczistaja. Józef berries contained a statistically similar amount of flavonols as Aromatnaja and Podarok Sadu, and these values were higher than the average flavonol content in the studied cultivars (637.53 mg/100 g DM). Teleszko et al. [12] reported that, among cultivars grown in Poland, Botaniczeskaja-Lubitelskaja berries had the lowest flavonol concentration (212.89 mg/100 g FW). The obtained results were in line with those published by Pop et al. [30], where the flavonol content ranged from 563 to 1437 mg rutin equivalent/100 g DM (for Serpenta and Tiberiu, respectively). However, the analyzed berries were richer in flavonols than those examined by Ma et al. [31], who reported a concentration from 23 (Oranzhevaya collected in China) to 250 mg/100 g (wild berries of ssp. *sinensis* from China). Variation in quantitative and qualitative flavonol profile occurs within subspecies and cultivars, and is affected by the harvest date, climatic, genetic, and geographic factors, transport, and storage [32,33].

### 3.3. Analysis of Carotenoids, Tocopherols, and Tocotrienols of Sea Buckthorn Cultivars

Carotenoids were classified as xanthophylls, carotenes, and esterified carotenoids. Their amounts for individual sea buckthorn cultivars are shown in Table 1. The total carotenoid concentration ranged between 46.61 mg and 508.57 mg/100 g DM, respectively, for Luczistaja and Aromatnaja. Józef berries were more than three times richer in carotenoids than Luczistaja and poorer than Aromatnaja. The values obtained were significantly higher than those observed for sea buckthorn fruits from Sweden [11] (from 11.99 mg to 142.49 mg/100 g DM) and growing in Romania [34] (from 53 mg to 97 mg/100 g DM).

Aromatnaja berries contained the most xanthophylls (80.73 mg/100 g DM). Nevertheless, Botaniczeskaja-Lubitelskaja and Luczistaja fruits were characterized by the highest percentage of these compounds with more than 74% of the total carotenoids. Moskwiczka and the new Józef berries contained similar amounts of xanthophylls and carotenes (about 41% of xanthophylls and 45% of carotenes). Fruits of cv. Aromatnaja contained 2 to 25 times more carotenes (225.42 mg/100 g DM) than other cultivars. Therefore, these berries were not yellow-orange but red (Figure 1). The remaining cultivars can be presented according to the increasing content of carotenes: Podarok Sadu > Józef > Moskwiczka > Botaniczeskaja-Lubitelskaja > Luczistaja. By comparison, in *H. rhamnoides* berries grown in Sweden, the carotene content was on average 29.66 mg/100 g DM [11]. On the other hand, research of Kruczek et al. [35] indicated that Aromatnaja and Moskwiczka berries were rich in carotenes (average 23.01 mg/100 g FW), and Botaniczeskaja-Lubitelskaja, Luczistaja, and Podarok Sadu contained less than 1 mg/100 g FW.

Esterified carotenoids were examined in four cultivars in the following order: Aromatnaja > Podarok Sadu > Józef > Moskwiczka. In the case of these berries, esterified carotenoids ranged from 12.11% to 19.80% of total carotenoids (for Moskwiczka and Aromatnaja, respectively). These contents were lower than in studies of Pop et al. [34] and Andersson et al. [11] in which esterified carotenoids accounted for an average of 71% and 55% of total carotenoids, respectively. Research on the sea buckthorn collected from Romania identified mono-esters and diesters of zeaxanthin and lutein esterified with palmitic, myristic, and stearic acid residues [34].

The lipophilic fraction of sea buckthorn berries also contains tocopherols and tocotrienols. However, the quantities determined were low and ranged between 27.12 mg of tocopherols and tocotrienols for Aromatnaja and 34.27 mg/100 g DM for Luczistaja (on average 29.03 mg/100 g DM). Cultivars grown in Sweden contained from 40.6 mg to 80.1 mg/100 g DM [6], and those from Finland contained tocopherols and tocotrienols from similar concentrations to almost four times higher [27].

### 3.4. Analysis of Fatty Acids of Sea Buckthorn Cultivars

In this research, omega-3, omega-6, omega-7, and omega-9 fatty acids were identified and divided into saturated fatty acids (SFAs) without C=C double bonds, monounsaturated fatty acids (MUFAs) with one such bond, and polyunsaturated fatty acids (PUFAs) with two or more double bonds between two connected carbon atoms. The fatty acid content in berries of sea buckthorn is summarized in Table 1.

Six fatty acids were identified in berries of the studied sea buckthorn cultivars, including two unsaturated acids (palmitic and stearic), two monounsaturated acids (palmitoleic and oleic), and two polyunsaturated acids (linoleic and linolenic). The dominant fatty acid was palmitic acid (C16:0), which ranged from 32.00% (Podarok Sadu) to 38.19% (Moskwiczka) of total fatty acid content. The berries were also abundant in palmitoleic acid (C16:1 n-7). Similar contents of oleic (C18:1 n-9) and linoleic acids (C18:2 n-6) as well as stearic (C18:0) and linolenic acids (C18:3 n-3) were determined. MUFAs dominated in the sea buckthorn berries (from 40.66% to 44.16%), except for cv. Moskwiczka in which saturated acids predominated (SFAs). PUFAs ranged from 16.11% (Moskwiczka) to 24.62% (Podarok Sadu). Generally, the fatty acid profile of the studied cultivars, including the newly bred cv. Józef, can be presented as mean 37.60% SFAs, 41.53% MUFAs, and 20.60% PUFAs. 

The results obtained were in line with those given for the analogous Aromatnaja, Botaniczeskaja-Lubitelskaja, Luczistaja, Moskwiczka, and Podarok Sadu, rich in palmitic, palmitoleic, and linoleic acids (up to 38.25%, 38.51%, and 14.11%, respectively) [12]. Yang and Kallio [36] observed high contents of palmitic, palmitoleic, oleic, and linoleic acids (up to 29.2%, 31.0%, 24.8%, and 33.9%, respectively) in oil from whole berries grown in Finland. In sea buckthorn berries grown in Romania, the main acids were oleic (up to 45.9%) and palmitic acids (up to 40.2%), in contrast with seeds rich in polyunsaturated acids [9,34,37].

Other studies also examined vaccenic acid (C18:1 n-7) in the sea buckthorn fruits in an amount of 4.5% to 9.8%, as well as myristic (C14:0), pentadecanoic (C15:0), hexadecanoic (C16:1 n-9), margaric (C17:0), arachidic (C20:0), and eicosenoic acids (C20:1 n-9) below 1% of the total amount of fatty acids [12,27,37].

### 3.5. Analysis of Biological Activity of Sea Buckthorn Cultivars: Anti-Oxidant, Anti-α-Amylase, Anti-α-Glucosidase, Anti-Lipase, and Anti-Lipoxygenase Effects

Table 2 presents anti-oxidant capacity measured by ABTS, FRAP, and ORAC assays in analyzed sea buckthorn fruits. According to all three methods, the highest anti-oxidant potential was observed for Aromatnaja berries, and the lowest for Luczistaja and Botaniczeskaja-Lubitelskaja. In the oxygen radical absorbance capacity (ORAC) test, the results ranged from 15.47 mmol to 34.68 mmol TE/100 g DM. The mean ABTS and FRAP activity values were 1.86 mmol and 2.59 mmol TE/100 g, respectively. Similarly, sea buckthorn berries harvested in China had ORAC activity from 26.6 to 36.9 mmol TE/100 g DM, in Turkestanica and Sinensis subspecies, respectively [7]. In line with Sharma et al. [38], the diversity of activity may result from the method (higher results in DPPH than ABTS test) and extraction because microwave application caused the highest activity of sea buckthorn berries in comparison to maceration, ultrasound, and Soxhlet. In addition, Gao et al. [39] reported that the reduction of ABTS activity during maturation correlated with decreasing concentrations of phenolic compounds and ascorbic acid. The lipid fraction activity increased due to the carotenoid synthesis, but this fraction did not significantly affect the anti-oxidant activity of berries. For example, fractions from ripe Aromatnaja berries had activities equal to 1.30, 0.45, and 0.56 mmol TE/100 g, for the phenolic, ascorbic, and lipophilic fractions, respectively.

Table 2 also shows anti-*α*-amylase, anti-*α*-glucosidase, and anti-lipase activity, as IC_50_ (mg/mL). The inhibitory activity against *α*-amylase ranged from 26.83 mg to 35.12 mg/mL (for Aromatnaja and Józef berries, respectively), while *α*-glucosidase inhibition was between 41.79 mg and 60.32 mg/mL (Botaniczeskaja-Lubitelskaja and Luczistaja, respectively). In all studied cultivars, *α*-amylase inhibition was stronger than that of *α*-glucosidase. In human trials, meals containing sea buckthorn berries reduced and delayed the postprandial insulin response and improved the glycemic profile [40,41]. Moreover, studies of Sharma et al. [38] and Xue et al. [42], carried out on rats and mice with type 2 diabetes, also confirmed the hypoglycemic effect of *H. rhamnoides* fruits. The inhibition toward pancreatic lipase in Aromatnaja and Moskwiczka berries was below 5.00 mg/mL. In the remaining cultivars, activity from 6.07 mg (Józef) to 14.02 mg/mL (Podarok Sadu) was recorded. The positive influence of sea buckthorn on lipid metabolism is confirmed by the examination of Linderborg et al. [43], according to which the addition of berries or their extract residues to meals delayed postprandial lipemia in humans.

The potential effect of sea buckthorn berries in relation to 15-lipoxygenase activity was analyzed, and the results were presented as the percentage of inhibition (at the concentration of 30 mg/mL) (Table 2). High anti-lipoxygenase activity of all cultivars ranged from 92.01% (Moskwiczka) to 100.00% (Aromatnaja and Podarok Sadu). Józef berries inhibited the enzyme activity equal to the others at 94.10%. The studied sea buckthorn berries can constitute a remedy in the concept of prevention and treatment of inflammatory diseases. Therefore, a good proposition is to use these fruits as a major component of functional foods. Zadernowski et al. [44] reported that lipophilic and ethanolic hydrophilic extracts from sea buckthorn decreased lipase activity, but the inhibition of lipoxygenase was higher than that of lipase.

### 3.6. Pearson’s Correlation and Principal Component Analysis (PCA)

Pearson’s correlation coefficients (r) between chemical composition and biological activity were determined. According to Rösh et al. [26], the dominant anti-oxidant compound in sea buckthorn is ascorbic acid, which is followed by flavan-3-ols and phenolic acids with catechol structures, in which the tested cultivars were not abundant. High correlations of vitamin C content with ABTS and FRAP anti-oxidant effects (*r* = 0.864 and 0.886, respectively) and between flavonols and the oxygen radical absorbance capacity (ORAC) (*r* = 0.617) were found. Carotenoids strongly influenced *α*-amylase inhibition (*r* = 0.747), lipoxygenase (*r* = 0.668), and antioxidant potential measured by ABTS, FRAP, and ORAC methods (mean *r* = 0.875). Carotenes and esterified carotenoids had a stronger effect on these activities than xanthophylls. SFAs correlated more strongly with the anti-lipase and anti-lipoxygenase potential (*r* = 0.601 and 0.710, respectively) than with antioxidant activity. Nevertheless, correlations of stearic acid were stronger with anti-oxidant, anti-*α*-glucosidase, and anti-lipase activity than for palmitic acid. However, oleic acid positively correlated with anti-*α*-glucosidase potential (*r* = 0.759). The correlation of linoleic and linolenic acids with the anti-lipoxygenase effect was positively moderate (*r* = 0.633). This could be due to the LOX function consisting in the catalysis of PUFAs.

Nutritionally, pectin is one of the soluble fiber fractions and may delay gastric emptying, which, in turn, is associated with reducing glycemic response [45]. There was, however, no correlation between pectins and biological activity, including α-amylase inhibition. Weickert and Pfeiffer [46] stated that a diet rich in insoluble dietary fiber in the form of cereals and whole grains may significantly reduce diabetes risk. However, there is no indisputable evidence that soluble dietary fibers from fruits and vegetables play a key role in this process, which may explain our results.

**Figure 2:**
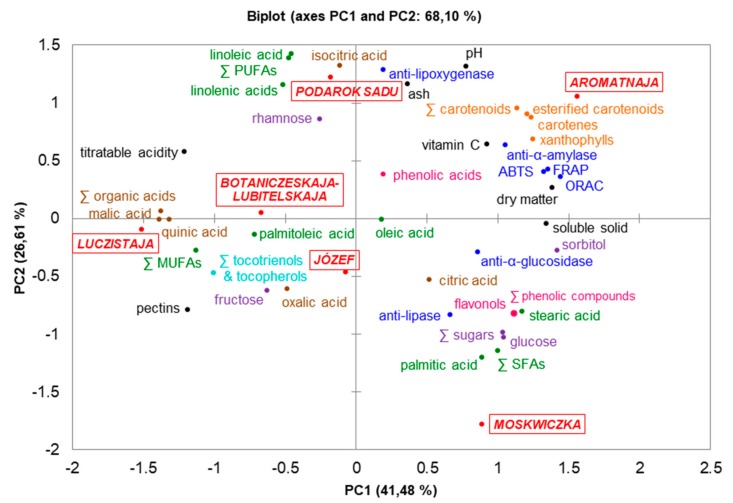
Principal component analysis biplot of chemical composition and biological effects of sea buckthorn cultivars. SFAs—saturated fatty acids. MUFAs—monounsaturated fatty acids. PUFAs—polyunsaturated fatty acids.

It should not be ruled out that the correlations between biological effects and lipophilic compounds may be apparent or false. The biological activities of sea buckthorn berries were tested on methanol-water solutions. Therefore, correlations for in vitro tests should be performed, with regard to lipophilic and hydrophilic compounds in *H. rhamnoides*. Other research on fruits also proved to be a selective correlation between chemical composition and biological activities. For example, Wang et al. [47] reported that flavonoids isolated from goji berries showed the most pronounced effect in scavenging free radicals (DPPH and ABTS) and chelating metal ions, while the zeaxanthin fraction was a strong scavenger of free hydroxyl radicals. In the studies of Wojdyło et al. [48], figs with a high content of sugars did not correlate with high anti-diabetic activity, but glycosylated derivatives of kaempferol, cyanidin, apigenin, polymeric procyanidins, and (−)-epicatechin correlated with the ability to inhibit *α*-amylase and *α*-glucosidase, and with the anti-oxidant effect (for the ORAC test). Ado et al. [49] found that a potent lipase inhibitor was kaempferol-3-*O*-rhamnoside isolated from *C. cauliflora* leaves. According to Stahl and Sies [50], carotenoids are effective anti-oxidants scavenging singlet molecular oxygen and peroxyl radicals. Principal component analysis (PCA) was conducted on the average contents of each chemical component (basic chemical composition, phenolic and carotenoid compounds, fatty acids), biological effects (anti-oxidant as ABTS, FRAP, and ORAC tests, enzyme inhibitory potential for *α*-amylase, *α*-glucosidase, lipase, and lipoxygenase), and berries of the tested *H. rhamnoides* cultivars. The outcomes are shown on the PCA biplot (Figure 2). The first two principal components (PC1 and PC2) explained 68.10% of the total variance (41.48% and 26.61%, respectively). The correlation biplot indicated the following: (1) Podarok Sadu berries were rich in PUFAs and had a strong anti-lipoxygenase effect, (2) anti-oxidant and anti-*α*-amylase activities were strongly correlated with the content of carotenoids and vitamin C, in which Aromatnaja fruits were particularly rich, (3) cv. Moskwiczka contained high concentrations of SFAs, phenolic compounds, and glucose, which, in turn, correlated with anti-lipase and anti-glucosidase activity, (4) Botaniczeskaja-Lubitelskaja, Luczistaja, and the new cv. Józef formed the most extensive berry cluster with high content of organic acids, MUFAs, tocotrienols, and tocopherols.

## 4. Conclusions

The study confirmed biochemical and functional differences among six cultivars of sea buckthorn berries cultivated in Poland. In conclusion, the analyzed sea buckthorn cultivars, including cv. Józef (reach in tocopherols and tocotrienols, MUFAs, palmitoleic acid (C16:1 n-7), fructose, quinic and oxcalic acids, and pectin) with biological potency against anti-*α*-glucosidase and anti-lipase activity, studied for the first time, can be a raw material in the development of innovative functional foods, nutraceuticals, and cosmeceuticals rich in chemical compounds with high biological activity. Research provides valuable information for selecting high-activity cultivars for further cultivation, and may also direct further *in vivo* testing for non-communicable diseases.

## Figures and Tables

**Table 1:** Basic chemical composition, phenolic compounds, carotenoids, tocopherols, and tocotrienols and fatty acids contents in sea buckthorn cultivars.

Components	Aromatnaja	Botaniczeskaja- -Lubitelskaja	Józef	Luczistaja	Moskwiczka	Podarok Sadu
Dry matter (%)	13.08 ± 0.23 a	11.88 ± 0.44 b	12.03 ± 0.38 b	11.78 ± 0.19 b	12.84 ± 0.34 ab	12.71 ± 0.40 ab
Soluble solid (°Bx)	7.2 ± 0.01 a	6.4 ± 0.00 a	7.1 ± 0.01 a	5.7 ± 0.01 a	7.2 ± 0.01 a	7.0 ± 0.00 a
pH	2.95 ± 0.01 a	2.90 ± 0.00 a	2.90 ± 0.01 a	2.90 ± 0.02 a	2.89 ± 0.00 a	2.93 ± 0.01 a
Titratable acidity (g malic acid/100 g) FW)	2.44 ± 0.01 c	2.62 ± 0.00 b	2.59 ± 0.08 b	2.71 ± 0.11 ab	2.48 ± 0.03 bc	2.79 ± 0.00 a
Ash (%)	0.43 ± 0.01 a	0.39 ± 0.01 a	0.40 ± 0.02 a	0.35 ± 0.04 a	0.31 ± 0.01 a	0.38 ± 0.05 a
Pectins (%)	0.34 ± 0.04 b	0.67 ± 0.16 a	0.21 ± 0.17 c	0.68 ± 0.18 a	0.64 ± 0.06 a	0.58 ± 0.18 ab
Vitamin C (mg/100 g FW)	158.81 ± 0.78 a	78.52 ± 0.64 b	64.92 ± 1.00 d	80.93 ± 2.32 b	71.32 ± 3.67 c	61.02 ± 0.21 d
Sugars (g/100 g FW)						
Rhamnose	0.05 ± 0.02 b	nd	0.04 ± 0.01 c	0.06 ± 0.00 a	nd	0.04 ± 0.02 c
Fructose	0.05 ± 0.00 d	0.04 ± 0.02 e	0.08 ± 0.02 b	0.10 ± 0.02 a	0.08 ± 0.02 b	0.07 ± 0.01 c
Sorbitol	0.14 ± 0.01 a	0.07 ± 0.00 c	0.11 ± 0.02 b	0.08 ± 0.00 c	0.13 ± 0.04 a	0.09 ± 0.02 bc
Glucose	1.96 ± 0.02 b	1.21 ± 0.02 d	1.73 ± 0.14 bc	1.49 ± 0.28 c	2.66 ± 0.65 a	1.29 ± 0.08 cd
∑ sugars	2.20 ± 0.01 b	1.34 ± 0.03 d	1.96 ± 0.19 c	1.70 ± 0.25 cd	2.87 ± 0.71 a	1.49 ± 0.13 cd
Organic acids (g/100 g FW)						
Oxalic acid	0.01 ± 0.00 b	0.01 ± 0.00 b	0.02 ± 0.00 a	0.02 ± 0.01 a	0.02 ± 0.02 a	0.02 ± 0.00 a
Citric acid	0.05 ± 0.00 b	0.01 ± 0.00 d	0.10 ± 0.05 a	0.03 ± 0.01 c	0.05 ± 0.01 b	0.02 ± 0.00 cd
Isocitric acid	0.21 ± 0.0 a	0.11 ± 0.00 a	0.02 ± 0.00 b	0.17 ± 0.01 a	nd	0.16 ± 0.00 a
Malic acid	0.96 ± 0.07 cd	1.95 ± 0.00 b	1.84 ± 0.09 b	2.87 ± 0.21 a	0.82 ± 0.03 d	1.17 ± 0.13 c
Quinic acid	0.09 ± 0.19 c	0.99 ± 0.03 a	0.27 ± 0.15 b	1.14 ± 0.22 a	0.07 ± 0.01 c	0.16 ± 0.09 c
∑ organic acids	1.33 ± 0.11 d	3.09 ± 0.27 b	2.25 ± 0.20 c	4.22 ± 0.13 a	0.96 ± 0.13 e	1.54 ± 0.14 d
Sugar: organic acid ratio	1.65 b	0.43 d	0.87 c	0.40 d	2.99 a	0.97 c
Phenolic compounds (mg/100 g DM)						
Phenolic acids	6.11 ± 1.98 c	5.18 ± 1.52 d	6.11 ± 1.88 c	5.46 ± 1.07 d	7.19 ± 2.52 b	8.94 ± 2.74 a
Flavonols	655.21 ± 46.16 b	484.22 ± 24.80 c	691.45 ± 56.36 b	463.14 ± 30.48 c	893.92 ± 54.96 a	637.22 ± 42.75 b
∑ phenolic compounds	661.32 ± 48.14 b	491.20 ± 26.71 c	697.56 ± 58.34 b	468.60 ± 31.55 c	901.11 ± 57.48 a	646.16 ± 45.52 b
Carotenoids (mg/100 g DM)						
Xanthophylls	80.73 ± 10.22 a	45.71 ± 4.62 cd	65.04 ± 5.33 b	37.76 ± 4.77 d	51.13 ± 7.18 c	60.27 ± 8.11 b
Carotenes	225.42 ± 12.27 a	16.03 ± 2.93 e	69.78 ± 4.60 c	8.85 ± 1.52 f	56.18 ± 4.27 d	115.62 ± 8.11 b
Esterified carotenoids	202.42 ± 7.02 a	nd	23.11 ± 2.04 c	nd	14.78 ± 1.35 d	70.37 ± 3.20 b
∑ carotenoids	508.57 ± 29.54 a	61.73 ± 7.55 e	157.93 ± 11.97 c	46.61 ± 6.29 e	122.09 ± 12.80 d	246.26 ± 19.42 b
∑ tocopherols and tocotrienols (mg/100 g DM)	27.12 ± 1.31 b	27.68 ± 1.42 b	28.23 ± 1.39 b	34.27 ± 2.00 a	29.29 ± 1.78 b	27.58 ± 1.55 b
Fatty acids (%)						
Palmitic (C16:0)	34.29 ± 0.01 b	33.24 ± 0.01 b	33.45 ± 0.01 b	32.82 ± 0.01 b	38.19 ± 0.01 a	32.00 ± 0.01 b
Palmitoleic (C16:1 n-7)	25.84 ± 0.02 b	23.67 ± 0.01 b	25.87 ± 0.01 b	31.25 ± 0.01 a	26.40 ± 0.01 b	26.17 ± 0.01 b
Stearic (C18:0)	4.14 ± 0.01 a	3.71 ± 0.01 ab	3.89 ± 0.01 a	2.65 ± 0.01 b	4.49 ± 0.01 a	2.72 ± 0.01 b
Oleic (C18:1 n-9)	14.90 ± 0.01 ab	17.83 ± 0.01 a	15.03 ± 0.01 ab	12.91 ± 0.01 b	14.81 ± 0.01 b	14.49 ± 0.01 b
Linoleic (C18:2 n-6)	17.42 ± 0.03 b	17.60 ± 0.01 b	16.74 ± 0.01 b	16.93 ± 0.01 b	13.16 ± 0.01 c	20.13 ± 0.01 a
Linolenic (C18:3 n-3)	3.43 ± 0.01 b	3.94 ± 0.01 ab	3.40 ± 0.01 b	3.44 ± 0.01 b	2.95 ± 0.01 c	4.49 ± 0.01 a
∑SFAs	38.42 ± 0.04 b	36.95 ± 0.02 c	37.34 ± 0.01 bc	35.47 ± 0.02 c	42.68 ± 0.02 a	34.71 ± 0.01 c
∑MUFAs	40.73 ± 0.05 b	41.50 ± 0.05 b	40.90 ± 0.06 b	44.16 ± 0.04 a	41.21 ± 0.04 b	40.66 ± 0.01 b
∑PUFAs	20.84 ± 0.03 b	21.54 ± 0.04 b	20.14 ± 0.01 b	20.37 ± 0.02 b	16.11 ± 0.01 c	24.62 ± 0.01 a

SFAs—saturated fatty acids. MUFAs—monounsaturated fatty acids. PUFAs - polyunsaturated fatty acids. The data shown are mean values ± SD (*n* = 3). nd - not detectable. DM—dry mass. FW—fresh weight. Different letters (a–d) in the same column denote a significant difference among varieties, according to Tukey’s test. *p* < 0.05.

**Table 2:** Anti-oxidant (mmol TE/100g DM), anti-α-amylase, anti- α-glucosidase, anti-lipase (IC_50_), and anti-lipoxygenase (percentage of inhibition) activities of sea buckthorn cultivars.

Properties	Aromatnaja	Botaniczeskaja- -Lubitelskaja	Józef	Luczistaja	Moskwiczka	Podarok Sadu
*Anti-oxidant activity*						
ABTS	3.58 ± 0.36 a	1.27 ± 0.10 d	1.12 ± 0.40 d	1.28 ± 0.01 d	2.22 ± 0.04 b	1.69 ± 0.12 c
FRAP	4.70 ± 0.14 a	1.84 ± 0.17 c	1.98 ± 0.12 c	1.85 ± 0.29 c	2.89 ± 0.09 b	2.29 ± 0.04 bc
ORAC	34.68 ± 2.14 a	18.41 ± 0.79 d	20.04 ± 0.62 c	15.47 ± 2.38 e	28.71 ± 0.41 b	27.30 ± 1.15 b
*Enzyme inhibitory activity*						
anti-*α*-amylase	26.83 ± 0.22 a	32.84 ± 0.09 c	35.12 ± 0.11 d	32.93 ± 0.48 c	29.62 ± 0.41 bc	28.49 ± 0.34 b
anti-*α*-glucosidase	44.45 ± 0.35 ab	41.79 ± 0.42 a	54.76 ± 0.72 c	60.32 ± 0.87 d	46.26 ± 0.31 b	58.89 ± 0.11 cd
anti-lipase	4.55 ± 0.16 a	9.20 ± 0.20 c	6.07 ± 0.19 b	10.07 ± 0.11 d	4.19 ± 0.17 a	14.02 ± 0.10 e
anti-lipoxygenase	100.00 a	92.22 d	94.10 c	97.43 b	92.01 d	100.00 a

The data shown are mean values ± SD (*n* = 3). *α*-amylase, *α*-glucosidase, and lipase inhibition are presented as IC_50_ in mg/mL, and anti-lipoxygenase effect as the percentage of inhibition (at the concentration of 30 mg/mL). DM—dry mass. Different letters (a–d) in the same column denote a significant difference among varieties, according to Tukey’s test. *p* < 0.05.
